# Voices of Women With Emergency Cesarean Section Experience: A Qualitative Approach

**DOI:** 10.7759/cureus.53429

**Published:** 2024-02-01

**Authors:** Eirini Orovou, Evangelia Antoniou

**Affiliations:** 1 Midwifery, University of Western Macedonia, Ptolemaida, GRC; 2 Midwifery, University of West Attica, Athens, GRC

**Keywords:** midwifery psychoeducation, birth trauma, delivery expectations, cesarean section, postpartum period

## Abstract

The midwifery psychoeducation, relationship with the midwives, feelings from the surgery, and delivery expectations are major factors that affect the birth experiences of mothers following an emergency cesarean section. This study aimed to give voice to mothers to express the feelings they had before, during, and after an emergency cesarean section and primarily to express whether their expectations were met after the surgery. The research was conducted on mothers who gave birth through an emergency cesarean section in a public hospital by completing specific questionnaires. This study was carried out with 15 mothers. The data was collected through a semi-structured questionnaire form, including socio-demographic characteristics. From the research, four main themes and 11 sub-themes emerged. The main themes described are "midwifery psychoeducation,” “relationship with the midwives,” “feelings from the C-section,” and “delivery expectations.” The majority of women did not attend parenting preparation classes either because they did not consider it necessary or because they were unaware of their existence. The presence of a midwife remains valuable during the perinatal period for most mothers. However, feelings vary between fear, shock, terror, disappointment, pain, and in some cases happiness. In the majority of cases, emergency cesarean section did not meet the expectations of mothers, who described it as a violent and sudden experience. The results highlight the need to strengthen midwives in order to promote psychosocial education, support during childbirth, and counseling for traumatic experiences.

## Introduction

A cesarean section, commonly known as a C-section, is a surgical procedure performed to deliver an infant. It involves making incisions in the abdominal wall and the uterus to remove the fetus [1]. This procedure is typically recommended when there are concerns about the safety or well-being of either the mother or the fetus during a vaginal delivery. The World Health Organization (WHO) has provided guidelines suggesting that the optimal rate of C-sections should be between 5% and 15% of all births [2]. This range ensures that C-sections are performed when necessary but avoids unnecessary surgical interventions. However, the rate of C-sections in Greece exceeds the WHO recommendations, since approximately 60% of Greek women give birth by C-section [3]. It is essential to address this high rate of C-sections in Greece and explore the reasons behind it. Factors such as cultural preferences, medical practices, fear of litigation, and lack of awareness about the risks and benefits of C-sections might contribute to this increase [4].

However, a C-section is not always a simple or enjoyable procedure due to the discomfort it brings, the sense of helplessness during childbirth, the time it takes for the body to heal, and the potential delay in bonding with the neonate [5]. Women who undergo C-section deliveries often feel anxious about the potential risks involved and harbor concerns about the extended recovery period and postoperative pain in comparison to a vaginal birth [6,7].

However, an emergency C-section has a higher morbidity rate for the mother compared to an elective or planned C-section [8-10]. Emergency C-section has also been associated with serious psychosocial consequences for the mother, such as reduced mother-child bond and breastfeeding problems, such as reduced duration or cessation of breastfeeding [11].

The type of anesthesia used in a C-section, and the women's expectations and experience of labor are all factors that can impact the satisfaction women derive from giving birth [12]. When women have a positive experience of labor, they tend to feel more in control, which in turn improves their relationship with their infants and the quality of care they can provide [13].

Pregnancy and birth experiences are unique to each woman and are interpreted differently [14]. They come with a set of expectations that women have regarding the birthing process. These expectations encompass their desires, actions, and thoughts concerning childbirth [15].

Factors affecting the expectation of childbirth can be influenced by various factors such as birth stories, experience of pain, concerns about infant harm or death, mother's beliefs, trust in midwives, knowledge about birth, and anxiety and loss of control [16].

It is essential to recognize that each mother's expectations and experiences after an emergency C-section can vary significantly. Communication, support, and access to accurate information can help address concerns and shape more realistic and positive expectations for childbirth [17]. Therefore, it is crucial to gain a thorough understanding of women's experiences during the early postpartum period. By determining their feelings, thoughts, and overall experiences about giving birth, healthcare providers can better tailor their care and support to meet the specific needs of each woman.

This research focuses on exploring and amplifying the perspectives of mothers who have undergone emergency C-sections. The objectives are to understand their delivery expectations and experiences during the procedure, the quality of care they received from the midwives, their feeling regarding the C-section, and their views on prenatal psychoeducation on C-section delivery. By giving voice to these mothers, the study aims to shed light on their unique perspectives and provide insights that can contribute to improving maternal care and support during emergency C-sections.

## Materials and methods

This study is a component of a broader investigation into birth trauma following C-section, and the overall research project is currently in progress. In this qualitative study, we utilized a descriptive study, which included semi-structured questions. Participants were selected based on a set of predetermined criteria for inclusion and exclusion. Specifically, participants had to be over 18 years old, have undergone an emergency C-section, have a sufficient understanding of the Greek language, and agree to participate in the research. Therefore, all participants were Greek-speaking mothers living in Greece, over the age of 18 who gave birth by emergency C-section in a Public Maternity Hospital in Athens from March to September 2023. All mothers who volunteered to participate in the study were fully informed about its objectives and they willingly consented to document their thoughts, with a guarantee of strict confidentiality.

For the needs of the research, questions suitable for the study were constructed. More specifically, the questions in the schedule were designed to explore various aspects related to the participants' experiences before and during their C-section. The main topics covered in the questions included pain management, the participant's relationship with the healthcare staff, any unmet expectations they may have had, and their experience of physical or mental preparation that may have occurred before their pregnancy. The questions ensured that all participants were asked the same set of questions, allowing for consistency and comparability in the responses (Table 1).

**Table 1:** Questions

Q1.1	Did you attend prenatal parenting preparation classes?
Q1.2	What was the reason you attended them?
Q1.3	What was the reason you didn't attend them?
Q1.4	How many prenatal education classes have you attended and in what format (online, face to face)?
Q2	What was your relationship with the midwives who took care of you during labor, cesarean section, and afterwards?
Q3	What feelings did the cesarean cause you?
Q4.1	Did the way you gave birth meet your expectations?
Q4.2	If they responded, explain why?
Q4.3	If they did not respond, explain why?

When conducting qualitative research, the sample size is not determined in advance. Instead, the saturation point determines the sample size. Saturation refers to the point at which no new information or themes emerge from the data, and participants' expressions become repetitive [18]. In this particular study, the sample size of 15 was determined based on reaching data saturation, where no new information or themes emerged and participants' expressions became repetitive. The socio-demographic and medical characteristics of the mothers are summarized in Table 2.

**Table 2:** Socio-demographic and medical characteristics of the participants (N=15) IUGR: intrauterine growth restriction.

Mother’s number	Age (years)	Family status	Education level	Type of previous deliveries	Complications during pregnancy	Gestational age (weeks)	Onset of labor	Causes for C-section
M1	27	Marriage	Junior High School	Primiparous	None	36	Automatically	Abnormal fetal rate
M2	35	Marriage	High School	Primiparous	None	39+2	Automatically	Failure to progress
M3	39	Marriage	High School	Cesarean	Preeclampsia	30	Automatically	Preeclampsia
M4	30	Marriage	High School	Vaginal	None	40+2	Induction	Abnormal fetal position
M5	25	Marriage	High School	Cesarean	Diabetes	39+3	Induction	Hydramnios
M6	31	Marriage	University	Vaginal	None	39+4	Automatically	Failure to progress
M7	30	Marriage	High School	Vaginal	None	40+2	Induction	Abnormal fetal rate
M8	34	Marriage	University	Vaginal	None	39	Automatically	Abnormal fetal position
M9	32	In relationship	University	Cesarean	IUGR	39	Induction	Failure to progress
M10	31	Marriage	High School	Primiparous	None	41	Automatically	Failure to progress
M11	27	Marriage	University	Primiparous	Diabetes	40	Automatically	Abnormal fetal rate
M12	38	Marriage	University	Primiparous	None	38+2	Induction	Failure to progress
M13	25	Marriage	University	Cesarean	None	38	Automatically	Inability to cooperate
M14	41	Marriage	University	Primiparous	None	40	Induction	Abnormal fetal rate
M15	31	Marriage	University	Primiparous	None	39	Induction	Failure to progress

Ethical approval was obtained from the Health Research Ethics Board at the Helena Venizelou-Alexandra Hospital (09/28-09-2022). Informed consent was obtained from the participants before the interview began, and the participants were given the option to stop the interview at any time and withdraw from the study. Responses were recorded using a specially designed open-ended and demographic questionnaire.

Data analysis

The interpretive content analysis method is a valuable approach for evaluating data. It is rooted in epistemologically interpretive or social constructivist perspectives, which emphasize that reality is constructed through human interaction. This analytical approach not only examines the manifest content of communication but also delves into the latent content, thereby uncovering the underlying factors that shape this construction process [19]. By using interpretive content analysis, researchers can go beyond mere statistical descriptions of interpersonal communication. This method allows them to gain insights into the goals and motivations of individuals involved in the communication process. Understanding these deeper aspects of communication can provide a more comprehensive understanding of the phenomenon under study [19].

The participant’s answers in our research were carefully read and transcribed by the researcher. The questionnaire data were repeatedly read by the researchers, following the method of interpretative content analysis. Similar answers were coded concurrently with the datasets (initial coding). After generating the codings, a visual map was constructed on paper to represent them. Each coding was assigned to a subcategory, and these subcategories were then grouped into a single main category. By combining these subcategories with other relevant categories, a comprehensive theme was formed.

After analyzing the data in this study, four main themes and 11 subthemes were identified (Table 3). These themes and subthemes were used to group and interpret the findings. By organizing the data in this way, it was easier to analyze and report the results. The main themes provide a broader overview of the findings, while the subthemes offer more specific details within each main theme. This approach helps to highlight the important patterns and trends that emerged from the data analysis process. It also adds structure and organization to the report, making it easier for readers to understand and follow the interpretation of the findings.

**Table 3:** The themes related to mother’s feelings

	Main themes	Subthemes
1.1	Midwifery psychoeducation classes	Opinion on parenting preparation classes
1.2.	Midwifery psychoeducation classes	Knowledge for midwifery psychoeducation classes
1.3.	Midwifery psychoeducation classes	Accessibility
2.1	Relationship with the midwives	Positive opinion
2.2.	Relationship with the midwives	Dissatisfaction
2.3.	Relationship with the midwives	Staff shortage
3.1	Feelings from the C-section	Feelings before the surgery
3.2.	Feelings from the C-section	Pain
3.3.	Feelings from the C-section	Experience after birth
4.1	Delivery expectations	Unfulfilled expectations
4.2	Delivery expectations	Fulfilled expectations

## Results

Four main themes were obtained from the study: “Midwifery psychoeducation” (Table 4), “relationship with the midwives,” “feelings from the surgery,” and “delivery expectations.” In these themes, women's views, feelings, expectations, and experiences are similar and different sometimes.

**Table 4:** Sub-Theme 1: Opinion on parenting preparation classes

Opinion on parenting preparation classes
I didn’t take parenting classes because I didn’t need them. I knew I could handle the situation (M1).
I postponed it again (Μ3).
I had participated in a previous pregnancy 2 years ago, so I had no reason to do it again... (Μ8).
I didn't want to attend classes (Μ12).
I chose not to attend classes because I didn't need them (Μ14).
The preparatory classes did not work for all mothers positively. Two mothers expressed their positive experiences.
I attended classes from the beginning of my pregnancy. They helped me to cope with my anxiety to some extent (Μ9).
I attended some classes. I think they helped me process the possibility of a C-section (M11).
Another mother expressed her unpreparedness for the unexpected surgery
I attended online lessons; however, I didn't feel prepared for the unexpected event that happened to me (Μ2).

The most notable observation regarding parenting classes is that many women were not informed about their presence and their importance. The lack of information for women concerns the location of the classes, their significant role in physical and mental preparation, and the importance of starting them early (Table 5).

**Table 5:** Sub-Theme 2: knowledge for midwifery psychoeducation classes

The lack of information
I didn't find the lessons useful then. I had not been informed by someone about their importance (M5).
I attended only 1 class. Unfortunately, I was informed late (M6).
I didn't know there were parenting preparation courses. If I had known, I would have watched them (M7).

One major issue that emerged was the free accessibility to parental preparation facilities in the province. The statements of mothers from the province revealed their dissatisfaction with this situation (Table 6). 

**Table 6:** Sub-Theme 3: Accessibility

The lack of accessibility
We don't have enough structures, unfortunately! (M10).
It was a long way from parenthood preparations structures. I am living in the countryside and I had no access (M13).
I live in a village and there were no free parenting classes (Μ15).

Main theme 2: Relationship with the midwives

The opinions of mothers about midwives and staff shortage issues were the main topics of this section (Table 7).

**Table 7:** Sub-Theme 1: Positive opinion

The positive role of midwives
I was very pleased with the midwives (M1).
I didn't have anyone with me! The midwife was there from the beginning to the end... I thank her! (Μ2).
I showed them trust and they helped me! (Μ3).
Very good all of them (Μ4).
Throughout this difficult process, luckily the midwives were extremely supportive (M6).
Great psychological support! (Μ9).
They helped me! (Μ14).
I showed trust and everything turned out well! (Μ15).

Midwife's work is recognized even by women who did not collaborate with her as they would have liked (Table 8).

**Table 8:** Sub-Theme 2: Dissatisfaction

The figure of the midwife during a difficult delivery is unique
I didn't have easy veins and they had to constantly poke me. They told me they understood, but I was in a lot of pain! They did everything so fast...(Μ5).
The midwives were very anxious.... They were coming to me infrequently. One of them apologized because I asked her to come repeatedly (Μ8).
In some cases, positive emotions from the mother's side balanced a negative behavior of the midwives, according to a mother
The midwives were a bit distant, but I had already had a C-section before. I knew the procedure (Μ12).
Midwives should defend women's rights during childbirth. Two mothers realize the importance of her role and assert them
I wanted to give birth naturally. They did not defend me as they should have! (M11).
They readily accepted the decision for a C-section. There are women like me who need more psychological support (Μ13).

Mothers seem to understand the problem of the lack of staff, but they feel alone (Table 9). 

**Table 9:** Sub-Theme 3: Staff shortage

Mothers expect midwives to be by their side
The midwives were very anxious.... They were coming to me infrequently. One of them apologized because I asked her to come repeatedly (M8).
They were all working without showing any particular interest in me. That day we were giving birth to mothers and the midwives were very few (M10).

Main theme 3: Feelings from the C-section

The birth process is a significant and transformative event in the lives of mothers, both physically and emotionally. In almost all cases here, the mothers were unprepared for a C-section. Emotions vary between fear, shock, terror, disappointment, pain, and in some cases happiness (Table 10).

**Table 10:** Sub-Theme 1: Feelings before the surgery

A C-section birth is sudden and violent, occurring with an immediacy that disrupts the mother's mental stability. Both fear and shock prevailed here
Very quick process with very quick decisions that I didn't have time to process (M6).
There was a state of panic.... I had lost control of the situation! (Μ13).
In a premature birth, a mother's fear for the baby's life is evident. In this case, a C-section appears as an unwanted surprise, very sudden and painful
I was 30 weeks. It was too early and I feared for the baby's life (M3).
I was very scared when they told me about the C-section... I didn't have time to realize it, but I quickly agreed. I wanted this to be over, what I was going through (Μ15).
A mother is unable to recall the moments before the surgery, displaying symptoms of psychological trauma
I don't remember anything! (Μ10).

The most commonly reported problems of mothers are severe pain and postoperative discomfort (Table 11). 

**Table 11:** Sub-Theme 2: Pain

Mothers describe the pain in detail and the transition from the pain of delivery to the pain of a C-section and recovery
I was in pain both during the delivery process and after the C-section (M11).
The incision is very painful. I can’t sit. I have to take painkillers all the time (M7).
Pain relief was an important topic in the discussion among the mothers. For a mother, general anesthesia was a failure, while for another mother, it was redemption from the shock of surgery and the pain
The spinal anesthesia didn't work and I could feel the pain. I ended up giving birth under general anesthesia and couldn't see the process (M4).
Luckily, I gave birth under general anesthesia, I couldn't bear to see and hear what was happening (M9).
The pain of vaginal delivery is described differently compared to surgery and the postoperative period. This shows that the pain does not stop with a C-section. A mother describes the pain before, during, and after a C-section
I had intense pain, possibly because I wasn't cooperating during contractions. During the surgery, they had to continue drilling me for blood and serum, and after the cesarean section, I continued taking painkillers (Μ5).

Most mothers describe their C-section experience as a sudden event to which they couldn't react. However, there are also mothers who try to balance between trauma and happy, attempting to experience the positive aspects of the C-section (Table 12). 

**Table 12:** Sub-Theme 3: Experience after birth

Mothers describe their C-section with raw narratives that reflect the violence and shock of the experience, using words that reveal psychological trauma
I hadn't prepared for a C-section. The surgery was shocking! (M2).
After the surgery I was in a lot of pain! I kept asking for painkillers. Who said C-section is easy? (M4).
Before I gave birth, the idea of a C-section did not scare me. I thought it didn't hurt as much as normal childbirth. I was disappointed of course..(M5).
I'm still processing the reasons for the emergency C-section the doctor told me. I think the C-section was unnecessary! (M8).
I was rushed to an emergency C-section because I couldn't cooperate. I think it was unnecessary….(M13).
For a mother, the memory of their previous C-section experience functioned protectively, as the process was familiar and the mother was prepared for what would happen to her.
I knew the C-section procedure; its repetition did not affect me psychologically (M12).
However, birth trauma can be well hidden behind the image of a healthy baby. Mothers may express a mix of feelings of disappointment and joy at the same time.
I suffered a lot.... Perhaps I could have had a vaginal birth, I don't know! What matters is that everything went well and the baby is healthy! (Μ14).

Main theme 4: Delivery expectations

The expectations of women for their childbirth consist of their desires, thoughts, and dreams related to giving birth. An emergency C-section is an unexpected event that finds the mother unprepared to face it. The unfulfilled desires are related to the failure of a vaginal birth and the violence of the urgent surgery. However, most mothers express their disappointment in the way they gave birth (Table 13).

**Table 13:** Sub-Theme 1: Unfulfilled expectations

Emergency C-section delivery is sudden and violent, occurring with an immediacy that disrupts the mother's mental calmness
I wanted to give birth naturally. I am disappointed! (Μ2).
No! I didn't expect it like this...(Μ4).
I am disappointed. I couldn't decide….(Μ5).
If I ever had to have a C-section in my life, I would want it to have been preceded by a natural birth (M8).
I wish I could give birth naturally (Μ9).
My wish was violated! It's not easy to manage all this...(Μ10).
…I think I could give birth normally (M14).
It was a bit scary as an experience (Μ15).
Vaginal delivery is the ideal birth method for mothers. Even the assisted vaginal delivery with vacuum extraction seems to be more preferable compared to an emergency C-section. Mothers hoped for something similar, but unfortunately in a surgical setting
My first delivery was vaginal with vacuum. I was hoping for something similar if it wasn't completely natural (Μ6).
I had a vaginal birth in my first child. I wanted the same this time (M7).
Mothers with previous experience of a C-section appear to be more disappointed. Their expectations of a vaginal birth are not fulfilled and give way to birth trauma
I was not adversely affected by the second C-section. The TOLAC may not have been successful, but the baby was fine (M12).
Throughout the pregnancy I prayed that the TOLAC would succeed. I had experience of a previous C-section and wanted it not to happen again. I was quite upset...(M13).
I had another C-section. I was now expecting to give birth normally. Unfortunately, it was repeated just as violently as the first time! (M3).

There was also a minority of women who felt that the C-section met their expectations (Table 14).

**Table 14:** Sub-Theme 2: Fulfilled expectations

Fulfilled expectations
The surgery went well, just as I had imagined (M1).
I wanted to give birth naturally, but after 3 days of suffering with no result, the C-section was a saving solution. Sometimes we have to appreciate the result (M11).

## Discussion

This qualitative study was aimed at understanding mother’s expectations and experiences during the procedure, the quality of care they received, their perceptions of pain, and their views on prenatal psychoeducation on C-section delivery. The main topics that are described were a) the midwifery psychoeducation, b) the relationship with the staff, c) feelings from the C-section, and d) delivery expectations.

The majority of mothers stated that they did not attend midwifery psychoeducation classes, either because they did not feel the need or because they had already experienced a vaginal or C-section delivery before. Various studies have reported that antenatal parenting classes have strengthened the parental role [20] and are related to decreased rates of C-sections [21].

Our results showed that many mothers were not informed about the usefulness of these classes during delivery. In contrast to our findings, a recent study conducted in Switzerland showed that mothers were aware of the importance of these classes in obstetric outcomes [22]. Not all mothers have access to face-to-face prenatal classes in rural areas. However, the importance of online monitoring of these classes is quite significant as it has beneficial effects on the mental and physical health of the mother [23].

Most mothers emphasized that the role of the midwife was important and helpful. However, it is understood that the staff shortage negatively affected the midwifery care provided to the mother [24]. In our research, some mothers expressed dissatisfaction with the care provided by midwives. The gap in communication between midwives and mothers leads to poor quality of care, deterioration of health outcomes, and potential dissatisfaction among mothers [25]. On the other hand, a strong relationship between a midwife and a mother could promote positive outcomes in childbirth and the postpartum period, especially in their mental health [26].

The experiences of mothers regarding the C-section were grouped into feelings before the surgery, the experiences of pain, as well as the post-surgery experiences. According to the responses, mothers' feelings before a C-section describe fear, shock, and a sense of loss of control. The fear of C-section, as expressed by mothers, has been described in other studies as well. Specifically, this fear arises from the sense of impending death for the life of the mother or the child [27].

Even though some mothers experienced pain before surgery, they continued to experience less pain during and, especially, after the procedure. The experience of pain in mothers after a C-section is common in similar studies [28] and seems to affect their mental well-being.

Women's feelings about their C-section are influenced by the presence of preparation, their experiences from previous births, and the outcome of the C-section. The majority of mothers in our study described the C-section as an unexpected and frightening experience. However, mothers who had given birth to their first child via C-section were more experienced and had more knowledge about C-section birth. According to previous research, mothers perceive an emergency C-section as a traumatic experience filled with negative emotions such as fear, guilt, or anger [29]. Recent studies, however, have highlighted the negative psychological reactions of an emergency C-section and the increased rates of psychological trauma in mothers [10].

From the responses of the mothers, it was found that mothers with a previous C-section expected a vaginal delivery, while mothers with a previous vaginal delivery hoped for a repeat vaginal delivery. The majority of narratives depict the dominance of disappointment, which appears submissive to a medical decision. Through the narrative, few mothers find the opportunity to position themselves by giving a positive meaning to the urgent surgery, and this is because the C-section, being a purely medical and surgical procedure that has no cultural meaning, is interpreted as an act of violence through an incision in the mother's body. Indeed, the literature suggests the risk of post-traumatic symptoms following an emergency C-section and we hypothesize that the echo of a traumatic birth experience could be compounded by the mother's previous life experiences [30].

This study has some strengths and limitations. One of the strengths of the study is that it is one of the few, to our knowledge, that has examined the experiences of women after an emergency C-section. Additionally, the study includes multiple thematic areas related to pregnancy, childbirth, and postnatal care, in order to draw safer conclusions regarding the causes of birth trauma. One limitation of the study is that it was conducted on a specific group of women, specifically those who had undergone an emergency C-section, and not other types of childbirth. However, emergency C-sections are considered a significant risk factor for mental trauma; therefore, we believe that these mothers are in greater need of intervention and counseling. Another limitation is that the research took place in a single hospital. However, this particular hospital is one of the largest public maternity hospitals in Athens.

## Conclusions

C-section is a real challenge for public health, as its rates are constantly increasing worldwide. Emergency C-section is experienced negatively by mothers, who are usually unprepared for this unexpected event. Insufficient mental and physical preparation of women for childbirth and the possibility of a C-section increase their vulnerability to the traumatic experience of giving birth. The figure of the midwife is unique during childbirth and has a dynamic role. Furthermore, the midwife can enhance a positive or negative experience for the mother, as she is part of the birthing process. To prevent mothers' prejudgments about childbirth, it is necessary to ensure an adequate number of midwives who will promote prenatal psychoeducation classes, remain by the woman's side during labor, alleviate trauma symptoms through counseling, and thus assist in the mother's psychological and physical recovery.

## Figures and Tables

**Figure 1:**
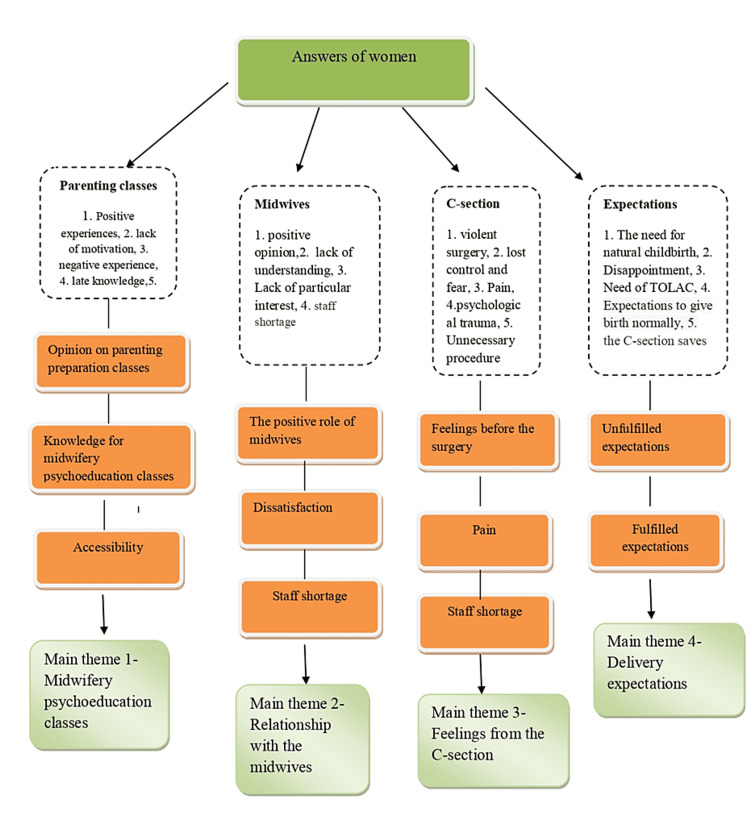
Visual map
